# Whole exome sequencing identified five novel variants in *CNTN2*, *CARS2*, *ARSA*, and *CLCN4* leading to epilepsy in consanguineous families

**DOI:** 10.3389/fgene.2023.1185065

**Published:** 2023-06-08

**Authors:** Angham Abdulrhman Abdulkareem, Qaiser Zaman, Hamza Khan, Sabar Khan, Gauhar Rehman, Nabeel Tariq, Mashal Ahmad, Muhammad Owais, Najumuddin, Osama Yousef Muthaffar, Fehmida Bibi, Rin Khang, Seung Woo Ryu, Muhammad Imran Naseer, Musharraf Jelani

**Affiliations:** ^1^ Department of Biochemistry, Faculty of Science, King Abdulaziz University, Jeddah, Saudi Arabia; ^2^ Center of Excellence in Genomic Medicine Research, King Abdulaziz University, Jeddah, Saudi Arabia; ^3^ Department of Zoology, Government Postgraduate College Dargai, Malakand, Khyber Pakhtunkhwa, Pakistan; ^4^ Higher Education Department, Peshawar, Khyber Pakhtunkhwa, Pakistan; ^5^ Department of Zoology, Abdul Wali Khan University, Mardan, Khyber Pakhtunkhwa, Pakistan; ^6^ Mardan College of Medical Technologies, Mardan, Khyber Pakhtunkhwa, Pakistan; ^7^ Programme of Biotechnology, Department of Applied Sciences, Faculty of Engineering, Science and Technology (FEST), Hamdard University, Karachi, Pakistan; ^8^ Department of Pediatrics, Faculty of Medicine, King Abdulaziz University, Jeddah, Saudi Arabia; ^9^ Special Infectious Agents Unit, King Fahd Medical Research Centre, King Abdulaziz University, Jeddah, Saudi Arabia; ^10^ Department of Medical Laboratory Technology, Faculty of Applied Medical Sciences, King Abdulaziz University, Jeddah, Saudi Arabia; ^11^ 3Billion Inc., Seoul, Republic of Korea; ^12^ Rare Diseases Genetics and Genomics, Centre for Omic Sciences, Islamia College, Peshawar, Khyber Pakhtunkhwa, Pakistan

**Keywords:** epilepsy, whole exome sequencing (WES), low income and middle income countries, molecular diagnoses, CNTN2, CARS2, ARSA, ClCN4

## Abstract

**Introduction:** Epilepsy is a group of neurological disorders characterized by recurring seizures and fits. The Epilepsy genes can be classified into four distinct groups, based on involvement of these genes in different pathways leading to Epilepsy as a phenotype. Genetically the disease has been associated with various pathways, leading to pure epilepsy-related disorders caused by *CNTN2* variations, or involving physical or systemic issues along with epilepsy caused by *CARS2* and *ARSA*, or developed by genes that are putatively involved in epilepsy lead by *CLCN4* variations.

**Methods:** In this study, five families of Pakistani origin (EP-01, EP-02, EP-04, EP-09, and EP-11) were included for molecular diagnosis.

**Results:** Clinical presentations of these patients included neurological symptoms such as delayed development, seizures, regression, myoclonic epilepsy, progressive spastic tetraparesis, vision and hearing impairment, speech problems, muscle fibrillation, tremors, and cognitive decline. Whole exome sequencing in index patients and Sanger sequencing in all available individuals in each family identified four novel homozygous variants in genes *CARS2*: c.655G>A p.Ala219Thr (EP-01), *ARSA*: c.338T>C: p.Leu113Pro (EP-02), c.938G>T p.Arg313Leu (EP-11), *CNTN2*: c.1699G>T p.Glu567Ter (EP-04), and one novel hemizygous variant in gene *CLCN4*: c.2167C>T p.Arg723Trp (EP-09).

**Conclusion:** To the best of our knowledge these variants were novel and had not been reported in familial epilepsy. These variants were absent in 200 ethnically matched healthy control chromosomes. Three dimensional protein analyses revealed drastic changes in the normal functions of the variant proteins. Furthermore, these variants were designated as “pathogenic” as per guidelines of American College of Medical Genetics 2015. Due to overlapping phenotypes, among the patients, clinical subtyping was not possible. However, whole exome sequencing successfully pinpointed the molecular diagnosis which could be helpful for better management of these patients. Therefore, we recommend that exome sequencing be performed as a first-line molecular diagnostic test in familial cases.

## Introduction

Epilepsy is a large and heterogeneous group of neurological disorders characterized by an imbalance or uncontrolled excitability of the brain, resulting in recurrent unprovoked episodes of epileptic seizures (Holmes and Ben-Ari, 2001; Stafstrom, 2006; Stafstrom and Carmant, 2015; Potnis et al., 2020). It is the second most prevalent neurological disorder after stroke, with an estimated prevalence of around 0.6%–1% of the global population, or approximately 50 million people. This means that out of every 100 people, about 1–1.5 people will have epilepsy (Stafstrom, 2006; Stafstrom and Carmant, 2015). Epilepsy is more likely to begin in children, accounting for about 65% of cases, or may affect people over 65 years of age. However, it can occur at any stage of life (Stafstrom and Carmant, 2015; Potnis et al., 2020). The epilepsy is classified in accordance with the 2017 ILAE (International Leagues Against Epilepsy) seizure classification, which is based on seizure (type, period), epilepsy (diagnosis, type, focal, generalized, combined), and syndrome (Rosenberg et al., 2016a; Scheffer et al., 2017; Scheffer et al., 2018).

Spasm is an abrupt, unintentional muscle contraction, seizure is a transient disruption in brain activity due to abnormal electrical activity, and epilepsy is identified by recurring seizures. The clinical features, severity, and further complications of epilepsy vary with gene, onset age, type, and the affected individual. The very first step in diagnosis is an episode of seizure, its intensity, duration, and recurrence. Seizures can cause a wide range of symptoms, from staring spells to violent convulsions, and can last from a few seconds to several minutes. Seizures can initiate focally and then generalize, resulting in epileptic spasms (Stafstrom and Carmant, 2015). A focal seizure originates in the neural network of one cerebral hemisphere, while a generalized seizure originates and spreads to the bilateral neural network of the cerebral hemisphere. A person is said to be epileptic when they have two or more unprovoked episodes of seizures (Stafstrom and Carmant, 2015; Scheffer et al., 2017; Scheffer et al., 2018). During an episode of seizure, an affected person may suddenly fall, experience convulsions and jerking of the partial or complete body without any clear reason. Their body becomes stiff, non-responsive, and they are unable to communicate. Their memory is temporarily blacked out, or they may feel confused in response. During a seizure, the affected person may have intermittent fainting spells with the loss of bowel or bladder control (Stafstrom, 2006; Kelemen et al., 2010; Hu et al., 2016; Kaculini et al., 2021). All the course of epileptic episodes are followed by extreme tiredness, panic, anger, or fear with no apparent reason. Peculiar changes in senses, such as smell, touch, and sound, are other prominent clinical phenotypes noted after epilepsy (Birbeck, 2000; Vazquez and Devinsky, 2003; Meldolesi et al., 2007; Kelemen et al., 2010; Mehdi et al., 2016).

The causes of epilepsy are diverse. Some are caused by brain injuries, strokes, infections, high fever, or tumors, and some have a genetic basis, while in some cases, the causes have never been determined. The broad causatives and diverse classifications make epilepsy one of the most difficult disorders to diagnose and treat (Marieb and Hoehn, 2007; Wang et al., 2017; Sarmast et al., 2020; Kaculini et al., 2021). To date, in the literature, a total of more than 977 genes have been reported to be associated with different forms of disorders that have epilepsy as a clinical feature. Among these, 84 are considered core epileptic genes, 73 are neurodevelopmental cum epileptic genes, 536 developmental malformations cum epileptic genes, and 284 as putative epileptic genes (Wang et al., 2017). The genes associated with epilepsy show autosomal dominant, autosomal recessive, X-linked, mitochondrial, as well as complex mode of inheritance patterns (Stafstrom, 2006; Stafstrom and Carmant, 2015; Wang et al., 2017).

In the presence of such diverse and overlapping clinical features, and a large number of known genes, diagnosing a patient with epilepsy is not an easy task. In the current study, we struggled to diagnose five families that are registered with mixed clinical features of seizures, mild to severe intellectual disabilities, ataxia, gait abnormalities, muscular anomalies, speech articulation, aggressive behavior, and neurodevelopmental complications (Table 1). In study, we selected a forward genetic analysis phenotypes to gene approach (Ali Khan et al., 2021), through whole exome sequencing (WES) (Umair et al., 2018; Seo et al., 2020; Zaman et al., 2023a; Umair, 2023) and confirming the segregation in the family through Sanger sequencing (Zaman et al., 2023a; Zaman et al., 2023b).

**TABLE 1:** A comparative table of the clinical phenotypes observed in the affected individuals of the five families (EP-01, EP-02, EP-04, EP-09, and EP-11) investigated this study.

S.No.	HPO standard entries	EP-01	EP-02	EP-04	EP-09	EP-11
1	Nystagmus (HP:0000639)	+	+	-	-	n/r
2	Intellectual disability (HP:0001249)	+	+	+	+	+
3	Memory impairment (HP:0002354)	+	+	+	+	+
4	Abnormal social behavior (HP:0012433)	+	+	+	+	+
5	Dysarthria (HP:0001260)	+	+	+	+	+
6	Hyporeflexia (HP:0001265)	n/r	+	+	n/r	+
7	Hypotonia (HP:0001252)	+	+	-	+	+
8	Gait ataxia (HP:0002066)	+	+	+	+	+
9	Cerebellar ataxia (HP:0001251)	n/r	+	+	n/r	+
10	Dystonia (HP:0001332)	+	+	-	n/r	+
11	Seizure (HP:0001250)	+	+	+	+	+
12	Causative gene	*CARS2*	*ARSA*	*CNTN2*	*CLCN4*	*ARSA*
13	OMIM	COXPD27	MLD	FAME5	MRXSRC	MLD

**Note:** “HPO” human phenotype ontology, “+” Phenotypes observed, “-” phenotypes not reported, “n/r” Phenotypes not recorded, “OMIM” online mendelian inheritance in man, COXPD27 (Combined Oxidative Phosphorylation Deficiency 27), MLD (Metachromatic Leukodystrophy), FAME5 (Familial Adult Myoclonic Epilepsy 5), MRXSRC (Raynaud-Claes Syndrome).

## Materials and methods

### Samples

**FIGURE 1:**
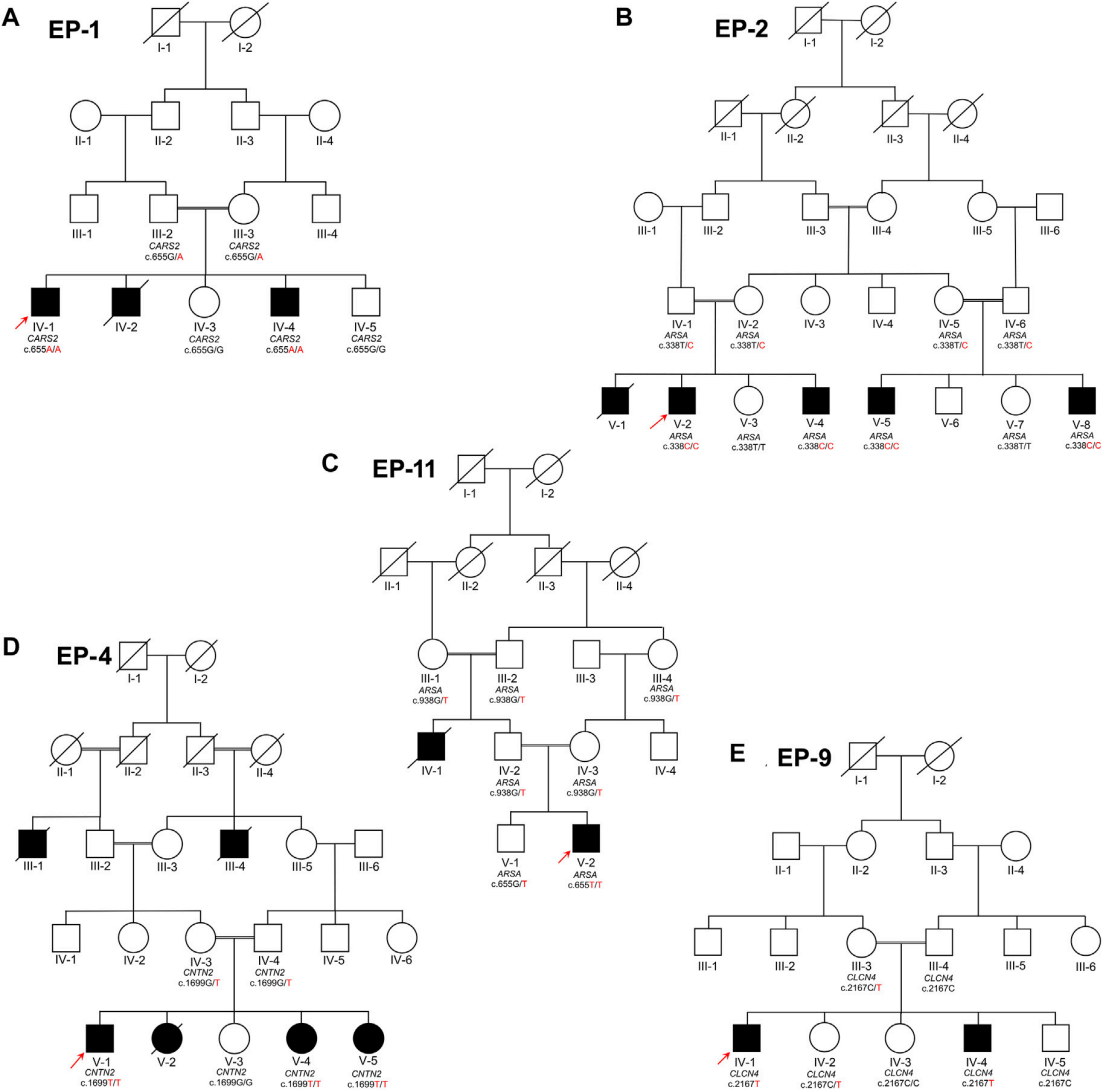
Five families labeled as EP-01 **(A)**, EP-02 **(B)**, EP-11 **(C)**, EP-04 **(D)**, and EP-09 **(E)**, each with indexed patients shown with an arrow, are shown. Family **(A)** has Combined Oxidative Phosphorylation Deficiency 27 as an autosomal recessive condition, while Families **(B)** and **(C)** have Metachromatic Leukodystrophy as an autosomal recessive condition. Family **(C)** also has Familial Adult Myoclonic Epilepsy 5 as an autosomal recessive condition and Family **(E)** has X-linked Raynaud-Claes syndrome. Sanger sequencing genotypes are written below symbols for samples available in each family.

Four families (EP-1, EP-2, EP-4, and EP-11), which segregate as autosomal recessive, while one family (EP-9) segregates as X-linked, were collected from the Khyber Pakhtunkhwa province of Pakistan. Informed written consent for the study was obtained from all affected individuals. Consent of under 18 years affected individuals was signed by the legal guardians of the families. The study was approved from the Institutional Bioethical Committee (IBC) of Islamia College Peshawar, with reference number 529/Oric/ICP, following guidelines of 2013 Helsinki’s Declaration (World Medical Association, 2013). The pedigrees of the families (Figures 1A–E) were drawn according to the standard protocol (Bennett et al., 2022). In each family, blood samples were collected from all available patients, their biological parents, and normal siblings in 5 mL K3EDTA tubes for genetic diagnosis. The blood samples were initially collected and stored on 4°C by the Department of Zoology, Government Postgraduate College Dargai Malakand, Khyber Pakhtunkhwa, and Pakistan.

### Genetic analysis

In each family, one index patient (EP-1 IV-1, EP-2 V-2, EP-4 V-1, EP-9 IV-1, and EP-11 V-2) with clinical phenotypes was chosen for whole exome sequencing, which was carried out by 3Billion Inc., (Seoul, Republic of Korea). DNA extraction was done using the QIAamp DNA Blood Mini Kit (QIAGEN, Venlo, Netherlands) with manufacture’s protocol. The exome, which covers around 22,000 genes that can cause diseases, was captured using the xGen Exome Research Panel v2 (Integrated DNA Technologies, Coralville, Iowa, United States) and sequencing was done using the NovaSeq 6000 (Illumina, San Diego, CA, United States). Over 9 billion bases were generated and aligned with the GRCh37 human genome database and the rCRS human mitochondrial genome reference database. The alignment resulted in a mean depth-of-coverage of 135 and the targeted bases were covered to a depth of ≥20x in almost 99% of the cases (Seo et al., 2020; Zaman et al., 2023a).

The in-house software, EVIDENCE at 3Billion Inc., (Seoul, Republic of Korea), was used to evaluate and prioritize genetic variations based on patient information, family background, and medical diagnosis, adhering to guidelines set by the American College of Medical Genetics and Genomics and the Association (ACMG) for Molecular Pathology (Biesecker and Harrison, 2018; Seo et al., 2020). Variants with a frequency higher than 5% were ruled out as per ACMG standards (Richards et al., 2015). Public databases such as gnomAD (https://gnomad.broadinstitute.org/), ExAC (https://gnomad.broadinstitute.org/), and 1000 genomes (https://www.internationalgenome.org/1000-genomes-browsers/index.html) were consulted to determine the variant’s occurrence in the general population. Databases of various diseases, including ClinVar (https://varsome.com) and UniProt (https://www.uniprot.org), HGMD (https://www.hgmd.cf.ac.uk/ac/) were also examined to assess the potential effect of the variants. *In silico* prediction of the variant was conducted with REVEL (Ioannidis et al., 2016) and 3CNet (Won et al., 2021).

Patients with rare genetic diseases have their clinical features transformed into standardized terms from the human phenotype ontology (https://hpo.jax.org/). These standardized terms are then compared to the symptoms of other 7,000 rare genetic diseases in databases such as OMIM (https://omim.org/) and Orphan Drugs (https://www.orpha.net/consor/cgi-bin/index.php). The selection of the causative variant, the specific genetic change responsible for the disease, is made based on the similarity score between the patient’s phenotype and the disease symptoms. The variants considered most likely to be the cause are prioritized based on ACMG guidelines.

### Sanger sequencing and segregation analysis

The variants identified in the index patients from each family were then analyzed within the family to confirm their segregation. The genomic DNA sequence of the *CARS2* (ENST00000375781.9), *ARSA* (ENST00000216124.10), *CNTN2* (ENST00000331830.7), and *CLCN4* (ENST00000380833.9) were downloaded from the online database Ensembl (https://asia.ensembl.org/ access on 15/06/2022). Primers were designed using Primer3plus (https://www.primer3plus.com access on 15/06/2022) (Supplementary Table S1). Sanger sequencing of all available affected individuals, their biological parents, phenotypically normal siblings, other family members, and at least 200 normal controls were performed by Macrogen Inc., Seoul, South Korea (https://dna.macrogen.com/ access on 06/07/2022).

### Protein modeling

The process of protein modeling described here involves the use of AI-based 3D structures of proteins, which were obtained from the UniProtKB database (https://www.uniprot.org/uniprotkb/ access on 15/01/2023). The structures of CARS2 (AlphaFold ID: AF8MVQ3-F1), ARSA (AlphaFold ID: AFA0A0C4DFZ2), and CLCN4 (AlphaFold ID: AF-Q4G0X3) were downloaded, as there were no known X-Ray crystallography-based 3D structures available (Jumper et al., 2021; Tunyasuvunakool et al., 2021). The 3D protein structures where needed, were predicted from the amino acid sequence using I-TASSER (Webb and Sali, 2016). The structures were then subjected to homology modeling using MODELLER, and energy minimization using AMBER ff99SB and UCSF Chimera 1.8.1 (Pettersen et al., 2004). Rotamers and amino acid outliers were removed using WinCoot 0.9.8.1, and the quality of the structure was evaluated using MolProbity and VERIFY3D (Eisenberg et al., 1997; Emsley and Cowtan, 2004). The optimized design was then visualized and analyzed using USCF Chimera 1.8.1 and PyMol to assess the structural and mutational effects. The pathogenicity and deleterious effect of the mutation in the protein was evaluated by using a computational tool MTBAN (Mutation Tolerance and Burden Analysis) (Kim et al., 2021a; Kim et al., 2021b).

## Results

### Clinical analysis

#### Family EP-01 CARS2

The family EP-1 has three affected siblings (IV-1, IV-2, IV-4), born to first cousin marriage in the fourth generation (Figure 1A). The two affected siblings, including the index (IV-1), died before attaining the age of 25 years, while other affected sibling is alive with mild phenotypes. The index patient died 6 months after the sampling. The phenotypes were noted to varies within the family and with age, and the late index patient had the most severe phenotypes of all. The affected individuals were born to normal mother via normal vaginal delivery, and no complications were noted at the time of birth. In the first decade of life, the index patient (IV-1) was reported with severe epileptic attacks and complications. As time passed, an increase in severity in the phenotypes was observed, including the appearance of strabismus, involuntary movements, nystagmus, visual anomalies, seizures, epileptic episodes, short memory loss, developmental regression, and areflexia. In childhood, the affected individuals sat and walked later, faced walking difficulties, ataxia, gait abnormalities, and walked on tiptoes. They appeared to have upper limb postural tremor, chorea, myoclonus, generalized dystonia, and severe muscular hypotonia. Postnatal microcephaly, mental deterioration, non-progressive intellectual disability, global developmental delay, and autistic behavior were also observed in the affected individuals. In the affected individuals, they did not develop proper language, had an absence of speech, and were noted to have feeding abnormalities and swallowing abnormalities like dysphagia.

#### Family EP-02 ARSA

The EP-02 is a large family with four alive (V-2, V-4, V-5, and V-8) and one deceased (V-1) affected individuals, in two clades (Figure 1B). The affected individuals were born to unaffected consanguineous union. The index patient (V-2) is a 6-year-old handicapped boy, born by normal vaginal delivery to normal mother. The disease phenotypes have a great diversity of severity within the family, and even within siblings. The index patient has the maximum phenotypes and is the most severely affected child among all the affected individuals. The affected individuals can be identified at the time of birth by their hyporeflexia, spastic tetraplegia, non-crying, and non-motile behavior. They have normal facial and skull morphology but are less active as compared to their normal siblings. By the age of 5 years, some of the affected individuals are still handicapped, unable to sit, stand, hold their body, or even hold something in their hands. Meanwhile, other affected individuals in the family are comparatively active, can sit, stand, and even walk, but they have delayed milestones, ataxia, unsteady disturbed gait, and walking difficulties. The index patient (V-2) was diagnosed with seizures, intellectual disability, mental deterioration, neurodegeneration, neurodevelopmental regression, leukodystrophy, periventricular white matter abnormalities, abnormality of the corpus callosum, and decreased nerve conduction velocity. They have feeding difficulties, uncontrolled urination, gallbladder dysfunction, cholecystitis, no speech development, no language development, dysarthria, nystagmus, vision abnormalities, and optic atrophy.

#### Family EP-11 *ARSA*

The family EP-11 has an affected index and his maternal uncle (IV-1 and V-2) (Figure 1C). Both the affected individuals were born to phenotypically normal parents, by normal vaginal delivery, and no birth complications were noted. They sat and walked on time, but near their second birthday, they progressively lost their grip, mobility, and standing capacity. In the index patient (V-1), the disease phenotypes appeared from 16 months, and till 21st month, he developed the maximum phenotypes. The index patient was initially reported with focal seizures and focal epilepsy. The episodes of epilepsy progressively increased, leading to paralysis, impaired walking, and muscular weakness throughout the body. The child lost the ability to walk, started to have an imbalanced gait and is now completely handicapped at the age of 2 years. The maternal uncle of the index patient, who was also born to cousin marriage, died at the age of 13 years, and was completely handicapped by the age of 2 years, like his nephew. Though he was not tested genetically, all the phenotypes and clinical diagnosis were similar to the genetically verified ones.

#### Family EP-4 *CNTN2*

The family EP-4 has three living (V-1, V-4, and V-5) and three deceased (III-1, III-4, and V-2) affected individuals (Figure 1D). All of the affected individuals were born to phenotypically normal parents through cousin marriages and no birth complications were recorded. In their early childhood, until the age of 7 and 8 years, all of the affected individuals were normal, but in their late first decade and early second decade, epilepsy attacks were recorded. The index patient (V-1) has the most severe disease phenotypes, but minor variations in phenotypic expression and severity were noted within age groups and within the family. According to clinical data, in late childhood, they were diagnosed with childhood epilepsy, generalized tonic-clonic seizures, focal-onset seizures, focal sensory seizures, generalized myoclonic seizures, myoclonus, and periodic paralysis with short-term memory loss. They have impaired memory, global developmental delay, mild intellectual disability. The affected individuals were unable to develop proper language, had difficulty with speech articulation, impaired and slurred speech. They have walking abnormalities, difficulty coordinating movements when walking, difficulty walking upstairs, muscle fibrillation, and tremor.

#### Family EP-9 *CLCN4*

The family EP-9 has two affected brothers (IV-1 and IV-4) and one moderately affected sister (IV-2), born to phenotypically normal parents through cousin marriage (Figure 1E). All the affected individuals exhibit hyper-emotional and aggressive behavior. They experience epilepsy, recurrent seizures, and focal myoclonic seizures, characterized by biting their teeth, tongue, and even causing self-inflicted injury to the tongue, followed by loss of consciousness. After 10–30 min, they wake up, but with temporary memory loss and complaints of muscle pain, fatigue, and laziness. They have unstable emotions, extreme aggression, and are intellectually disabled, with poor memory and low intelligence. They haven’t developed proper speech, only able to say a few phrases in a slurred tone and have speech articulation issues. One of the sisters has moderate phenotypes, but sometimes experiences more rapid onset of epilepsy, seizures, memory loss, and is less aggressive.

### Genetic analysis

#### *CARS2* (OMIM: 612800)

**FIGURE 2:**
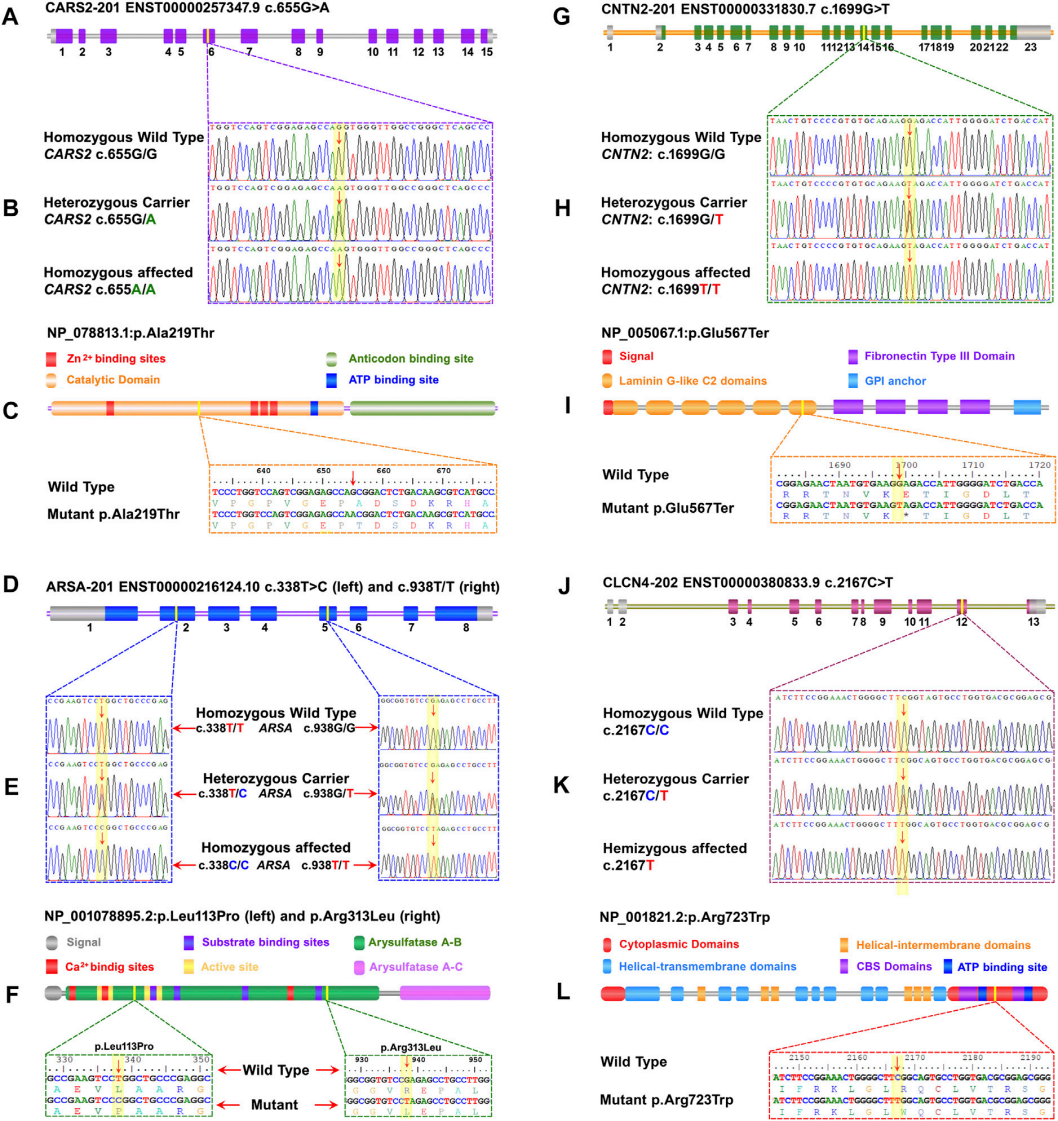
A schematic representation of the four genes with exons and focus point of mutation, trace files for mutant and carrier, as well as wild-type, linear protein with different parts, and single-letter translation for both wild and mutant proteins are shown in each panel. Panels **(A)** to **(C)** represent *CARS2*, **(D)** to **(F)** represent *ARSA*, **(G)** to **(I)** represent *CNTN2*, and **(J)** to **(L)** represent *CLCN4*. **(A)** The *CARS2* has 15 coding exons, and the point of mutation is in exon 6. In panel **(B)**, the homozygous affected, heterozygous carrier, and homozygous wild type for *CARS2* **(C)**655G>A are shown. Panel **(C)** displays the Cysteinyl-tRNA Synthetase 2 protein with a key for its structure and active site. The mutation p.Ala219Thr is located in the cytoplasmic domain of the functional unit. **(D)** The *ARSA* has 8 coding exons and two different mutations in two different families. The **(C)**338T>C of family EP-02 is located in exon 2 and the **(C)**938G>T of family EP-11 is located in exon 5. In panel **(E)**, the homozygous affected, heterozygous carrier, and homozygous wild-type for ARSA **(C)**338T>C of family EP-02 and **(C)**938G>T of family EP-11 are shown. Panel **(F)** displays the Arysulphatase A enzyme with a key for its structure and active site. The mutations p.Leu113Pro (Family EP-02) and p.Arg313Leu (Family EP-11) are located in the B domain of the Arysulphatase A enzyme. **(G)** The *CNTN2* has 15 coding exons, and the point of mutation is in exon 14. In panel **(H)**, the homozygous affected, heterozygous carrier, and homozygous wild type for *CNTN2* **(C)**655G>A are shown. Panel **(I)** displays the Contactin 2 protein with a key for its structure and active site. The mutation p.Glu567Ter is located in the sixth laminin G-like C2 domain. **(J)** The *CLCN4* gene has 13 total exons, and the point of mutation is in exon 12. In panel **(K)**, the homozygous affected, heterozygous carrier, and homozygous wild type for *CLCN4* **(C)**2167C>T are shown. Panel **(L)** displays the ClC-4 protein, a member of the CLCN family of voltage-dependent chloride channels, with a key for its structure and active site. The mutation p.Arg723Trp is located in the functional cytoplasmic domain of the ClC-4 protein.

A pathogenic biallelic variant (c.655G>A) was identified in *CARS2* (NM_024537.3) that causes Combined oxidative phosphorylation deficiency 27 (COXPD27, OMIM: 616672) in an autosomal recessive pattern. This variant leads to an amino acid change (p.Ala219Thr) in the catalytic domain of the CARS2 protein (NP_078813.1) (Figures 2A–C).

#### *ARSA* (OMIM: 607574)

Two families (EP-02 and EP-11) have biallelic variants in *ARSA* (NM_001085426.2), linked to Autosomal Recessive Metachromatic Leukodystrophy (MLD, OMIM: 250100). c.338T>C in EP-02 and c.938G>T in EP-11 are VUS with predicted effects on p.Leu113Pro and p.Arg313Leu in the B domain of Arylsulfatase-A enzyme (NP_001078895.2) (Figures 2D–F **)**. *In-silico* predictions show potential damaging effects on the gene or gene product (REVEL: 0.99; 3Cnet: 0.99/REVEL: 0.98; 3Cnet: 1.00 respectively).

#### *CNTN2* (OMIM: 190197)

A pathogenic biallelic variant (c.1699G>T) was identified in *CNTN2* (NM_005076.5), linked to Autosomal Recessive Epilepsy Myoclonic Familial Adult Type 5 (FAME5, OMIM: 615400). The variant is predicted to result in a nonsense mutation (p.Glu567Ter) in the 6th laminin G-like C domain of the CNTN2 protein (NP_005067.1) (Figures 2G–I), leading to loss of normal protein function via nonsense-mediated mRNA decay.

#### *CLCN4* (OMIM: 302910)

A hemizygous variant (c.2167C>T) of uncertain significance was identified in *CLCN4* (NM_001830.4), linked to Raynaud-Claes syndrome (OMIM: 300114). This variant is predicted to cause a missense mutation (p.Arg723Trp) in the cytoplasmic domain of the CLCN4 protein (NP_001821.2) (Figures 2J–L). *In-silico* predictions show potential damaging effects on the gene or gene product (REVEL: 0.87; 3Cnet: 0.62).

### Proteins pathogenicity analysis

**FIGURE 3:**
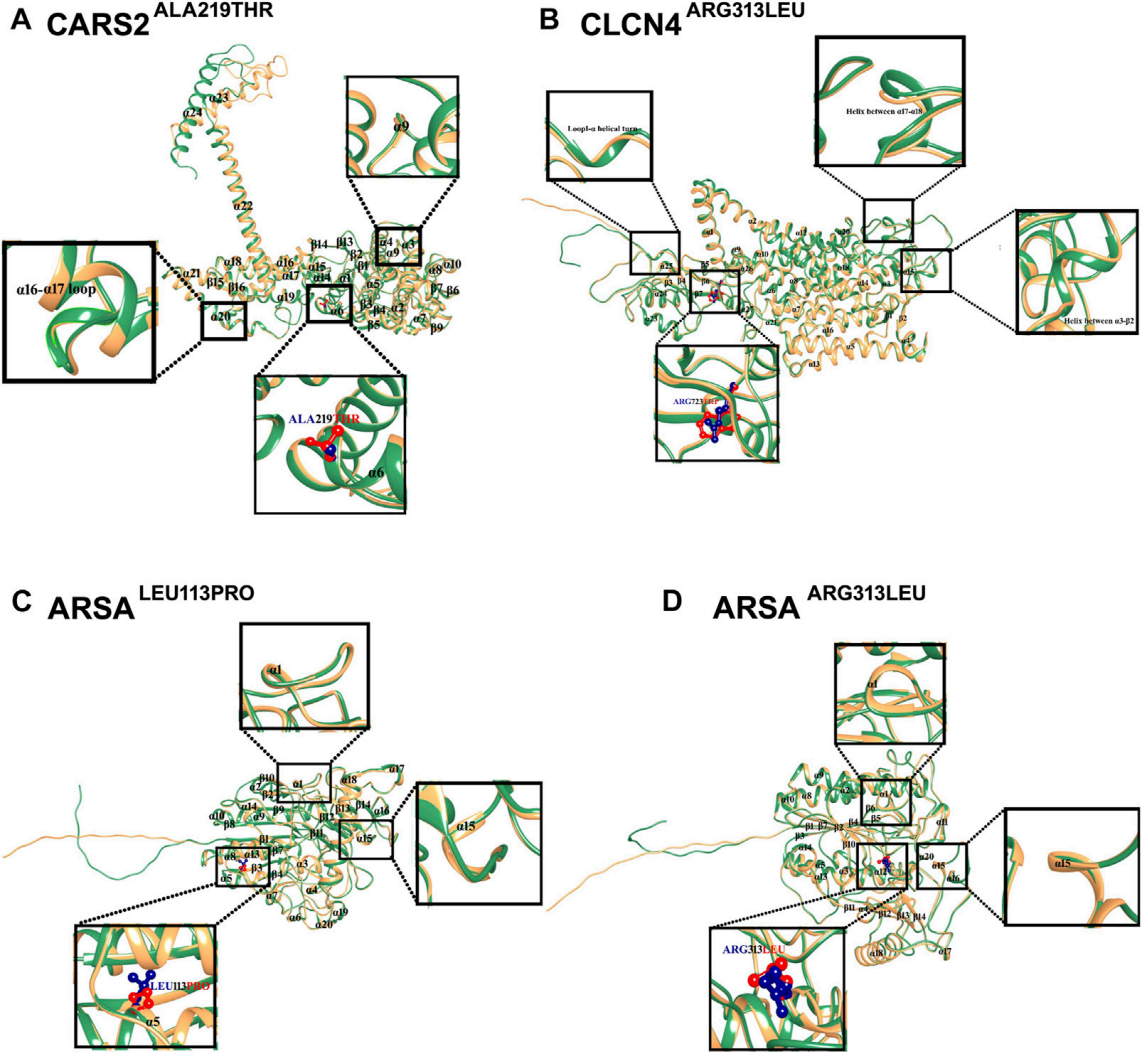
Mutational and Structural Analysis of CARS2, ARSA, and CLCN4. The 3D structures of the wild-type forms of CARS2, ARSA, and CLCN4 are displayed in sandy brown, while the mutated forms are displayed in sea green. Blue represents the wild-type amino acids, and red represents the mutated amino acids. Secondary structures are labeled in black, and the structural changes and mutations are depicted in the inset. **(A)** Superimposition of CARS2^WT^ (sandy brown) and CARS2^ALA219THR^ (sea green) shows the structural differences due to mutations in CARS2. **(B)** Superimposition of CLCN4^WT^ (sandy brown) and CLCN4^ARG313LEU^ (sea green) illustrates the structural differences due to mutations in CLCN4. Blue represents ARG, while red represents TRP. **(C)** Superimposition of ARSA^WT^ (sandy brown) and ARSA^LEU113PRO^ (sea green) highlights the structural differences due to mutations in ARSA. Blue represents LEU, while red represents PRO. **(D)** The structural differences due to mutations in ARSA are shown through the superimposition of ARSA^WT^ (sandy brown) and ARSA^ARG313LEU^ (sea green). Blue represents ARG, while red represents LEU.

The structural analysis of predicted CARS2, ARSA, and CLCN4 proteins revealed high stability and reliability in most regions, with pLDDT values between 90 and 70. This indicates a stable and reliable structure crucial for proper functioning and interaction with other molecules. 3D Structural analysis of CARS2^WT^ and CARS2^ALA219THR^ showed the wild type has 24 α-helices and 16 β-sheets, with the mutation located at α6. The comparison revealed that α9 became a loop, a tri-residual (Threonine-Glycine-Glutamine) α-helical turn was induced between α16-α17 loop, and the C-terminal moved about 45° and outward (Figure 3A). The wild type CLCN4^WT^ has 17 α-helices and 7 β-sheets, with the mutation at β6. The comparison with CLCN4^ARG723TRP^ showed an α-helical turn was induced in loop1 between α3-β2 and an α-helix between α17-α18 (Figure 3B). For ARSA^WT^ and ARSA^LEU113PRO^/ARSA^ARG313LEU^, the wild type has 20 α-helices and 14 β-sheets. ARSA^LEU113PRO^’s mutation is at α-5, and ARSA^ARG313LEU^’s at α12. The comparison showed a tri-residual (Glycine-Cysteine-Tyrosine) α-helical turn was induced between β-1 and α-1 in the loop, and α-15 became a loop in both mutations (Figures 3C, D).

## Discussion

The current project enrolled five families, including 12 living and 6 deceased individuals with a range of developmental delay, intellectual disability, walking difficulties, gait issues, muscle weakness, and epilepsy phenotypes. Our primary aim was to reach an accurate diagnosis for these individuals, given the varied and overlapping phenotypes. To achieve this, we used forward genetic analysis through whole exome sequencing, and, with *in-silico* analysis, protein mutagenesis and segregation analysis through Sanger sequencing, we were able to diagnose these families to the best of our expertise.

The Combined Oxidative Phosphorylation Deficiency 27 (OMIM: 616672) disorder is characterized by a range of symptoms, as exhibited by the first family (EP-01), ranging from severe muscle weakness, delayed development, seizures, epilepsy, to autistic behavior (Hallmann et al., 2014; Coughlin et al., 2015). The disorder’s underlying cause has been linked to mutations in *CARS2* (OMIM: 612800), encoding Cysteinyl-tRNA Synthetase 2, a key component in protein synthesis. *CARS2* is located on chromosome 13q34 and consists of 7 exons, encoding a potential member of class I aminoacyl-tRNA synthetase enzymes (Coughlin et al., 2015). The *CARS2*-encoded protein is located in the nucleus but thought to be transported to the mitochondria, where it participates in charging cysteine to tRNA (Coughlin et al., 2015). Despite its critical role in protein synthesis, only a limited number of mutations have been reported to cause COXPD27, including 8 missense, 1 splicing, and a small deletion (Wu et al., 2022). We have identified a likely pathogenic biallelic variant, CARS2 c.655G>A, changing p.Ala219Thr in the Cysteinyl-tRNA Synthetase 2 protein’s catalytic domain (NP_078813.1). Though, the variant is lying in the less conserved region of CARS2 protein but has been previously linked to severe myoclonic epilepsy, progressive spastic tetraparesis, vision and hearing impairment, and cognitive decline (Hallmann et al., 2014). Our patient also had symptoms of severe muscle weakness, microcephaly, intellectual deterioration, non-progressive intellectual disability, global developmental delay, and autistic behavior. *In-silico* predictions indicate this variant disrupts pre-mRNA splicing (RF: 0.976≥0.6, ADA: 1.0≥0.6). The variant’s significance is further confirmed by protein pathogenicity analysis and the mutant alleles family segregation.

**FIGURE 4:**
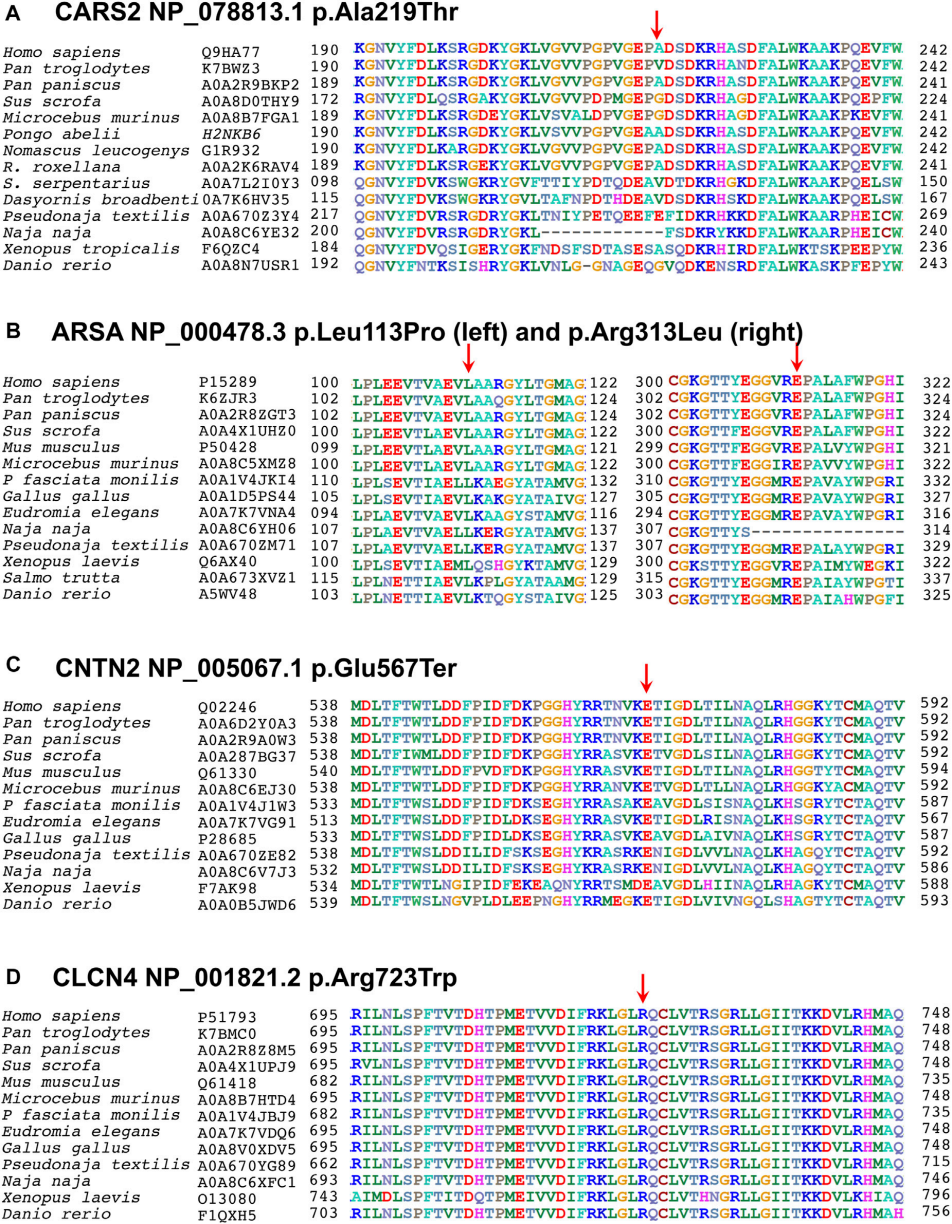
Multiple sequence alignments of CARS2 (Q9HA77), ARSA (P15289), CNTN2 (Q02246), and CLCN4 (P51793) are shown, downloaded from UniProtKB. The first column shows species, and the second column shows their UniProtKB ID. The numbers before and after the sequences indicate the corresponding amino acids, with an arrow above indicating the mutated point. **(A)** In Cysteinyl-tRNA Synthetase 2 protein, the alanine at codon 219 is replaced by threonine in a less conserved region, but in-silico predictions show that this variant disrupts pre-mRNA splicing. **(B)** In Arysulphatase A enzyme, the leucine at codon 113 is replaced by proline (left family EP-02), and the arginine at codon 313 is replaced by leucine (right family EP-11), both mutations lying in the highly conserved Arysulphatase-A B domain. **(C)** In CNTN2 protein, the glutamic acid at codon 567 is replaced by a termination codon in a highly conserved region of the first domain. **(D)** In CLCN4, the arginine at codon 723 is replaced by leucine in a highly conserved region of the cytoplasmic topological domain.

Metachromatic leukodystrophy (OMIM: 250100) is a genetic disorder caused by mutations in the *ARSA* (OMIM: 607574) that result in deficiency of the arylsulfatase A enzyme. This enzyme cleaves sulfatides, a common sphingolipid in myelin that is crucial for the differentiation of oligodendrocytes and the formation of paranodal regions. The arylsulfatase A enzyme acts in the lysosome to hydrolyze sulfate groups of sulfatides (Honke et al., 2002; Shaimardanova et al., 2020). Its deficiency causes sulfatide accumulation in the central and peripheral nervous system and other tissues, leading to progressive demyelination and a range of neurological symptoms, such as growth retardation, delayed motor development, seizures, epilepsy, and muscle spasms, as seen in families EP-09 and EP-11 (Honke et al., 2002; Ługowska et al., 2009; Rosenberg et al., 2016b; Shahzad et al., 2017; Wang et al., 2019; Shaimardanova et al., 2020; Xu et al., 2021). The *ARSA* (OMIM: 607574) is located on chromosome 22q13.33 and consists of 8 exons that encode the 489 amino acid ARSA protein, a member of the lysosomal arylsulfatase enzyme family (Honke et al., 2002; Shaimardanova et al., 2020). To date, over 322 mutations in the ARSA gene have been reported to cause metachromatic leukodystrophy, including 237 missense, 18 splicing, 31 small deletions, 20 small insertions, 6 small insertions/deletions, 6 gross deletions, 1 gross insertion, and 2 complex rearrangements, mostly affecting the C (alpha)-formylglycine. These mutations reduce sulfatase activity and lead to the accumulation of sulfatides in the brain, causing destruction of myelin-producing cells and impairing the nervous system in metachromatic leukodystrophy (Shaimardanova et al., 2020; Amr et al., 2021; Eckhardt, 2008; von BuÈlow et al., 2001). In our study, we report variants c.338T>C in family EP-02 and c.938G>T in family EP-11 in the ARSA gene (NM_001085426.2) which result in changes in the amino acids p.Leu113Pro and p.Arg313Leu in the B domain of the Arylsulfatase-A enzyme (NP_001078895.2), respectively. These variants are categorized as having uncertain significance (VUS) and are located in the highly conserved B domain region of the Arylsulfatase-A enzyme (Figure 4B). *In silico* predictions, conservation analysis, and protein modeling indicate that the variants are damaging to the protein’s structure and function (REVEL: 0.987≥0.6; 3CNET: 0.985≥0.75). Our findings suggest that these mutations are likely to result in autosomal recessive Metachromatic Leukodystrophy, exhibiting symptoms similar to those reported, with the possibility of minor variations among our patients (Shahzad et al., 2017; Shaimardanova et al., 2020; Amr et al., 2021).

Familial Adult Myoclonic Epilepsy 5 (OMIM: 615400) is a rare genetic form of epilepsy that manifests in adulthood and is characterized by myoclonus, jerky, involuntary muscle twitches or movements, and seizures (recurrent episodes of abnormal electrical activity in the brain that cause changes in behavior, movement, sensation, or consciousness). However, there is disagreement regarding FAME5’s relation to intellectual or memory decline or other neurological symptoms, such as cognitive impairment, in contrast to other forms of epilepsy (Stogmann et al., 2013; Striano et al., 2013). Nevertheless, we are reporting it in our patients. In addition to generalized seizures and evolved into focal seizures and neurodevelopmental impairment, speech articulation difficulties, walking abnormalities, inability to coordinate movements when walking, difficulty walking upstairs, muscle fibrillation, and tremor were also observed in our patient. FAME5 is caused by biallelic mutation in the *CNTN2*, which is located on chromosome 1q32.1 and consists of 23 exons. The gene produces a 7916 bp transcript and encodes a 1040 amino acid protein called contactin 2, which is expressed by neurons and myelinating glial cells. Contactin 2 is a neuronal cell adhesion molecule in the contactin subgroup of the immunoglobulin superfamily and plays a crucial role in organizing juxtaparanodal regions in myelinated fibers (Stogmann et al., 2013; Striano et al., 2013). It works together with contactin-associated protein-like 2 to form a scaffold that accumulates voltage-gated potassium channels at the juxtaparanodes, which are crucial for maintaining membrane potential and modulating action potential frequency during repetitive firing. To date, 13 mutations in the *CNTN2* have been associated with epilepsy and FAME5, including 7 missense mutations, splice variants, and 2 small deletions (Chen et al., 2021). In the present study, we have identified a missense homozygous variant *CNTN2* c.1699G>T in 3 diseased individuals of the affected family EP-4, that leads to a change in the amino acid p.Glu567Ter in the 6th laminin G-like C domain of the CNTN2 protein (NP_005067.1).

The affected members of family EP-09 display a mixture of moderate to severe phenotypes, including intellectual disabilities, psychiatric disorders, brain abnormalities, impaired language development, behavioral problems, seizures, epilepsy, progressive ataxia, and facial dysmorphisms as reported to be Raynaud-Claes syndrome. The Raynaud-Claes syndrome is a type of X-linked intellectual disability and was first reported by Martine Raynaud and his team in 1996 (Raynaud et al., 1996). The was reported to cause by hemizygous mutation in *CLCN4* gene. The *CLCN4* is located on chromosome Xp22.3 and encodes a 760 amino acid protein known as ClC-4, which is part of the CLCN family of voltage-dependent chloride channels (Nguyen et al., 2011). Although the exact role of the gene is unknown, it may function as an electrogenic 2Cl-/H+ exchanger and play a role in the development of neurological disorders such as Raynaud-Claes Syndrome (van Slegtenhorst et al., 1994; Veeramah et al., 2013a; Jentsch and Pusch, 2018). So far, 36 mutations have been reported in the *CLCN4*, consisting of 30 missense mutations, 1 splicing mutation, 1 small deletion, 1 gross deletion, and 1 gross insertion, which are associated with Raynaud-Claes Syndrome (Elliott et al., 2022). In this study, a hemizygous variant c.2167C>T, predicted to change the amino acid p.Arg723Trp in the cytoplasmic domain of the ClC-4 protein (NP_001821.2) (Figure 2H) of uncertain significance, was identified in 2 diseased brothers of the affected family EP-09 with clinical presentations such as epilepsy, periods of seizures, memory loss, intellectual disability, dull memory, speech problems, and highly aggressive behavior. More or less similar phenotypes were also documented by Veeramah (2016) and Hu (2013), who also reported variations in the *CLCN4* in patients affected by Raynaud-Claes Syndrome (Veeramah et al., 2013b; Hu et al., 2016).

## Conclusion

Epilepsy is a clinical phenotype that can be linked to a diverse clinical pathogenicity and intricate group of genetic disorders, encompassing over 950 identified genes (Wang et al., 2017). For accurate and efficient diagnosis, it is hard to rely solely on clinical investigations due to the overlapping features found in several subtypes. WES has the ability to rule out not only previously known or reported variants but it also has the potential to identify novel variants. As compared to targeted genes panels WES has more coverage and may be used as a first line molecular diagnostic tool in families from Pakistan. Studies have indicated that cousin marriages account for over 60% of disease allele segregations in Pakistani societies (Umair et al., 2018; Zaman et al., 2023a; Zaman et al., 2023b). To reduce the burden of disease alleles, we recommend the strict implementation of premarital testing, carrier screening, and newborn genetic testing in societies such as Pakistan (Alyafee et al., 2021; Alyafee et al., 2022; Zaman et al., 2023a; Zaman et al., 2023b).

## Data Availability

The datasets presented in this study can be found in online repositories. The names of the repository/repositories and accession number(s) can be found in the article/Supplementary Material.
