# Supplementary material for: Whole exome sequencing identified five novel variants in CNTN2, CARS2, ARSA, and CLCN4 leading to epilepsy in consanguineous families
Source: Front Genet. 2023 Jun 8;14:1185065. doi: 10.3389/fgene.2023.1185065 (PMC10285458; doi:10.3389/fgene.2023.1185065)
Supplement: Supplementary file 1 [file Table1.DOCX]

**Supplementary Table 1.** Primer list with gene name, exon No., annealing temperature, and product size. For primer design, the genomic DNA sequences of the genes were retrieved from the Ensembl genome browser (https://asia.ensembl.org/)

| **No** | **Gene** | **Exon No** | **Primer (**5′ 🡪 3′) | **Product Size** | **Tm** |
| --- | --- | --- | --- | --- | --- |
| 1 | *CNTN2* | 4 | F 5′-TGGTTCTAAGTGATGAGTCGTGA-3′ | 615 | 60 |
|  |  |  | R 5′-TCACAGAGGCTGTCACATGG-3′ |  |  |
| 2 | *CARS2* | 6 | F 5′-CTGAGCCAAGGAGTTCAAGG-3′ | 842 | 60 |
|  |  |  | R 5′-TGTTTGATTCCCTTAGACAGTATCC-3′ |  |  |
| 3 | *ARSA* | 2 | F 5′-TCTCTCTAGGGAAGGCCACA-3′ | 560 | 59 |
|  |  |  | R 5′-CATGTAGCGGGCCTCTAGTC-3′ |  |  |
| 4 | *ARSA* | 5 | F 5′-CGGGCAAGCTTTGTGACTTA-3′ | 565 | 60 |
|  |  |  | R 5′-CCACTTGGATGCCACTCAG-3′ |  |  |
| 5 | *CLCN4* | 12 | F 5′-AGCTGCAGAGAGATGCCAAT-3′ | 470 | 59 |
|  |  |  | R 5′-TTCTTCAGGGCTGAGACCAT-3′ |  |  |

**Table 1:** A comparative table of the clinical phenotypes recorded in the affected individuals of the families.

| S/No | Phenotype / Family | EP-01 | EP-02 | EP-11 | EP-04 | EP-09 |
| --- | --- | --- | --- | --- | --- | --- |
| 1 | Nystagmus | + | + | n/r | - | - |
| 2 | Mental Abnormalities | + | + | + | + | + |
| 3 | Memory abnormalities | + | + | + | + | + |
| 4 | Abnormal Behaviour | + | + | + | + | + |
| 5 | Speech abnormalities | + | + | + | + | + |
| 6 | Decrease Reflex responses | n/r | + | + | + | n/r |
| 7 | Muscles Tune (Hypotonia) | + | + | + | - | + |
| 8 | Walking abnormalities | + | + | + | + | + |
| 9 | Gait disturbance | + | + | + | + | + |
| 10 | Ataxia | n/r | + | + | + | n/r |
| 11 | Involuntry Muscular Movement (Dystonia) | + | + | + | - | n/r |
| 12 | Seizures | + | + | + | + | + |
| 13 | Epilepsy | + | + | + | + | + |
| 14 | Memory loss after Epileptic Episode | + | + | + | + | + |
| 15 | Causative Gene | *CARS2* | *ARSA* | *ARSA* | *CNTN2* | *CLCN4* |
| 16 | Disoder | COXPD27 | MLD | MLD | FAME5 | MRXSRC |

**Note:** “+” Phenotypes observed, “-” Phenotypes not reported, “n/r” Phenotypes not recorded, COXPD27 (Combined Oxidative Phosphorylation Deficiency 27), MLD (Metachromatic Leukodystrophy), FAME5 (Familial Adult Myoclonic Epilepsy 5), MRXSRC (Raynaud-Claes Syndrome).
