# Supplementary material for: Whole exome sequencing identified five novel variants in CNTN2, CARS2, ARSA, and CLCN4 leading to epilepsy in consanguineous families
Source: Front Genet. 2023 Jun 8;14:1185065. doi: 10.3389/fgene.2023.1185065 (PMC10285458; doi:10.3389/fgene.2023.1185065)
Supplement: Supplementary file 2 [file Table2.DOCX]

**Supplementary Table 2:** Prediction tools for variants

ARSA-201 ENST00000216124.10 c.338T>C

ACMG Classification as Pathogenic **15** points =**15**P-**0**B, ClinVar **Likely Pathogenic,** conservation score phyloP100: **7.271**

| **Pathogenic** | **Likely Pathogenic** | **Uncertain Significance** |
| --- | --- | --- |
| **182** | **92** | **253** |

CARS2-201 ENST00000257347.9 c.655G>A

ACMG Classification **Uncertain Significance 5**points =**6**P-**1**B, Conservation Scores phyloP100: **4.941**

7 pathogenic (6 missense, 1 frameshift), 2 likely pathogenic

CNTN2-201 ENST00000331830.7 c.1699G>T

ACMG Classification

**Pathogenic**

**11** points =**11**P-**0**B

Conservation ScoresphyloP100: **9.345**

| **Pathogenic** | **Likely Pathogenic** | **Uncertain Significance** |
| --- | --- | --- |
| **8** | **6** | **261** |

CLCN4-202 ENST00000380833.9 c.2167C>T

ACMG Classification

**Likely Pathogenic**

**7**points =**7**P-**0**B

Conservation Scores

phyloP100: **2.993**

| **S. No** | **Engine** | **ARSA-201 c.338T>C** | | | **CARS2-201 c.655G>A** | | | **CNTN2-201 c.1699G>T** | | | **CLCN4-202 c.2167C>T** | | |
| --- | --- | --- | --- | --- | --- | --- | --- | --- | --- | --- | --- | --- | --- |
|  |  | **Calibrated Prediction** | **Score** | **Indicative Prediction** | **Calibrated Prediction** | **Score** | **Indicative Prediction** | **Calibrated Prediction** | **Score** | **Indicative Prediction** | **Calibrated Prediction** | **Score** | **Indicative Prediction** |
| 1 | ACMG Classification | Likely Pathogenic | 15 points =15P-0B | ------ | Uncertain Significance | 5 points =6P-1B | ------ | Pathogenic | 11 points =11P-0B | ------ | Likely Pathogenic | 7points =7P-0B | ------ |
| 2 | Conservation Scores by phyloP100 | ------ | 7.271 | ------ | ------ | 4.941 | ------ | ------ | 9.345 | ------ | ------ | 2.993 | ------ |
| 3 | BayesDel addAF | Pathogenic | 0.4927 | Damaging | Benign | -0.3662 | Tolerated | Pathogenic | 0.625 | Damaging | Pathogenic | 0.5525 | Damaging |
| 4 | BayesDel noAF | Pathogenic | 0.5799 | Damaging | Benign | -0.6584 | Tolerated | Pathogenic | 0.66 | Damaging | Pathogenic | 0.5558 | Damaging |
| 5 | MetaLR | Pathogenic | 0.9801 | Damaging | Benign | 0.07349 | Tolerated | ------ | ------ | ------ | Pathogenic | 0.8828 | Damaging |
| 6 | MetaRNN | Pathogenic | 0.9859 | Damaging | Benign | 0.1682 | Tolerated | ------ | ------ | ------ | Pathogenic | 0.9559 | Damaging |
| 7 | MetaSVM | Pathogenic | 1.0512 | Damaging | Benign | -1.067 | Tolerated | ------ | ------ | ------ | Pathogenic | 0.9912 | Damaging |
| 8 | REVEL | Pathogenic | 0.987 | ------ | Benign | 0.119 | ------ | ------ | ------ | ------ | Pathogenic | 0.87 | ------ |
| 9 | BLOSUM | Uncertain | -7 | ------ | Uncertain | -1 | ------ | ------ | ------ | ------ | Uncertain | -7 | ------ |
| 10 | DANN | Uncertain | 0.9989 | ------ | Benign | 0.9254 | ------ | Uncertain | 0.9972 | ------ | Pathogenic | 0.9993 | ------ |
| 11 | dbscSNV | ------ | ------ | ------ | Pathogenic | 0.9997 | ------ | ------ | ------ | ------ | ------ | ------ | ------ |
| 12 | DEOGEN2 | Uncertain | 0.5985 | Damaging | Benign | 0.1285 | Tolerated | ------ | ------ | ------ | Pathogenic | 0.8686, | Damaging |
| 13 | EIGEN | Pathogenic | 0.8753 | ------ | Benign | -0.0951 | ------ | Pathogenic | 0.9184 | ------ | ------ | ------ | ------ |
| 14 | EIGEN PC | Pathogenic | 0.7384 | ------ | Benign | 0.09593 | ------ | Pathogenic | 0.7736 | ------ | ------ | ------ | ------ |
| 15 | FATHMM | Pathogenic | -5.13 | Damaging | Benign | 1.4 | Tolerated | ------ | ------ | ------ | Uncertain | -2.6 | Damaging |
| 16 | FATHMM-MKL | Uncertain | 0.9627 | Damaging | Uncertain | 0.9542 | Damaging | Pathogenic | 0.9901 | Damaging | Uncertain | 0.939 | Damaging |
| 17 | FATHMM-XF | Pathogenic | 0.9659 | Damaging | Uncertain | 0.7031 | Damaging | Benign | 0.3032 | Neutral | ------ | ------ | ------ |
| 18 | LIST-S2 | Pathogenic | 0.9818 | Damaging | Benign | 0.6242 | Tolerated | ------ | ------ | ------ | Pathogenic | 0.9986, | Damaging |
| 19 | LRT | Uncertain | 9.9999e-7 | Deleterious | Benign | 0.9111 | Neutral | ------ | ------ | ------ | Pathogenic | 0 | Deleterious |
| 20 | M-CAP | Pathogenic | 0.6178 | Damaging | Pathogenic | 0.3968 | Damaging | ------ | ------ | ------ | Pathogenic | 0.5729 | Damaging |
| 21 | Mutation Taster | Uncertain | 1 | Disease causing | Benign | 0.8979 | Disease causing | ------ | ------ | ------ | Uncertain | 1 | Disease causing |
| 22 | PrimateAI | Uncertain | 0.7331 | Tolerated | Benign | 0.3849 | Tolerated | ------ | ------ | ------ | Pathogenic | 0.9088 | Damaging |
| 23 | PROVEAN | Pathogenic | -6.39, -5.76 | Damaging | Benign | -0.83 | Neutral | ------ | ------ | ------ | Pathogenic | -6.99, | Damaging |
| 24 | SIFT | Pathogenic | 0.001 | Damaging | Benign | 0.664 | Tolerated | ------ | ------ | ------ | Pathogenic | 0 | Damaging |
| 25 | SIFT4G | Pathogenic | 0.002, 0.001 | Damaging | Benign | 0.572 | Tolerated | ------ | ------ | ------ | Pathogenic | 0 | ------ |
